# Antibiotic Therapies in Maxillofacial Surgery in the Context of Prophylaxis

**DOI:** 10.1155/2015/819086

**Published:** 2015-02-01

**Authors:** Bogusława Orzechowska-Wylęgała, Adam Wylęgała, Michał Buliński, Iwona Niedzielska

**Affiliations:** ^1^Department of Cranio-Maxillofacial Surgery, Medical University of Silesia, Francuska 20/24, 40-027 Katowice, Poland; ^2^Department of Internal Medicine and Oncology, Medical University of Silesia, Reymonta 8, 40-027 Katowice, Poland

## Abstract

*Objectives*. There is no single pattern for preventive action as to the duration and type of antibiotic therapy in maxillofacial surgery. In these circumstances, it appears reasonable to set relevant standards for prophylactic procedures after such surgeries. *Methods*. Retrospective analysis of bacteriological tests has been carried out as well as a susceptibility evaluation of cultured bacterial and fungal strains to antibiotics over a five-year period in subjects treated at the Cranio-Maxillo-Facial Clinic in Katowice. A total of 726 bacterial and fungal strains were cultured in 484 patients (200 women and 284 males). The age of the patients was 40.2 on average. *Results*. The most frequent bacteria isolated from the patients were Gram-positive 541 (74.5%). Gram-negative bacteria were present in 177 (24.4%) cases. Fungi of the *Candida* genus were isolated in eight cases (1.1%). *Conclusions*. The most often isolated bacteria were *Streptococcus mitis* and * Streptococcus oralis*, whose number has grown over the last two years. Empiric therapies should be based on ciprofloxacin and gentamicin. It has been observed that all the Gram-positive bacteria are becoming more resistant to all antibiotics. Ampicillin and imipenem were antibiotics with the steepest resistance reduction while vancomycin showed the lowest resistance drop.

## 1. Introduction

Each surgical intervention in the facial skeleton results in bacteria dissemination into the blood. Disturbing dermal integuments or breaking the epithelial continuity within the oral cavity leads to the penetration of the patient's body by microorganisms [1]. If the patient has a resistant surgery it may lead to a passing bacteraemia, yet in particular, long procedures like oncological, post trauma or orthognatic surgeries will always be a test for the immunological resilience of the body. Postoperative complications may have the form of inflammations of both soft and hard tissues [2, 3]. In order to prevent them, antibiotics and chemotherapeutic agents are commonly used, a practice known as empiric antibiotic utilisation for prophylactic purposes, which fails to be therapeutically successful given the drug resistance of bacterial strains [4]. An empirically administered antibiotic should have a broad spectrum of action against most pathogens [3]. It will then, however, change the bacterial flora of the host, which becomes resistant to the action of the antibiotic drug. Consequently, antibiotics with broad and narrow spectrum have the same therapeutical effect bar the unfavourable impact of the former on the physiological flora and growth of resistant bacteria [5]. Another key issue is such dosage selection as to ensure that the drug concentration in the plasma does not drop below the minimum inhibitory concentration (MIC), and the ratio between the peak concentration (Cmax) and the MIC must be appropriate, too. Antibiotics are often recommended in a routine and irrational manner (on the patient's request) particularly in the case of viral conditions or fever of unknown origin, which contributes to the development of an ever-growing number of resistant and multidrug-resistant strains [6]. Patients treated with therapeutic doses can develop superinfections or new infections with, for instance, intestinal* Pseudomonas* bacteria or mycoses of the digestive tract and the respiratory or urogenital systems. The condition is a result of the drug's inhibitory action on the healthy bacterial flora which produces antibacterial substances. The broader the spectrum of action and the longer the time of use, the higher the risk of such a superinfection [7]. A major problem is the cross-resistance, that is, when a microorganism acquires resistance to several groups of antibiotic drugs. Moreover, a wrongly used antibiotic can result in allergic or toxic responses as well as impact drug interactions [8]. Apart from the correct use of antibiotics, another essential factor is appropriate drug dosing. When drugs are not correctly dosed and selected, the risk of hospital-acquired bacteremia increases, particularly its critical variety. That may take place when, after a few days of taking antibiotics and after momentary improvement, the patient's general condition deteriorates including fever incidence [9].

The recommendation concerning the use of antibiotics in perioperational prophylaxis developed for the Ministry of Health fails to provide detailed information on maxillofacial surgeries. Likewise, there is no single pattern for preventive action as to the duration and type of antibiotic therapy in maxillofacial surgery. In such circumstances, it appears reasonable to set relevant standards for prophylactic procedures after such surgeries.

This paper aims to answer the following questions.

1. What have been the dominant pathogens over the 2 years?
2. Over the few years, has the bacterial flora changed (e.g., whether the number of* Pseudomonas* or* Acinetobacter*, etc., is growing)?
3. What is the susceptibility of the dominant pathogens to antibiotics and has that changed over the years?
4. Which antibiotic should be used preventively so as to preclude postsurgery inflammatory complications?

To that end, a retrospective analysis has been carried out of bacteriological tests as well as a susceptibility evaluation of cultured bacterial and fungal strains to antibiotics over a five-year period in subjects treated at the Cranio-Maxillo-Facial Surgery Chair and Clinic and the Clinical Outpatient Unit for Maxillofacial Surgery in Katowice.

## 2. Material and Methods

A total of 726 bacterial and fungal strains were cultured in 484 patients (200 women and 284 males) treated at the Cranio-Maxillo-Facial Surgery Chair and Clinic and the Clinical Outpatient Unit for Maxillofacial Surgery based at the Independent Public Clinical Hospital (hereinafter “SPSK-M”) in the Polish city of Katowice between 1 January, 2008, and 31 December, 2012 (Table 1). The age of the patients was between 8 and 82 years (40.2 on average). The material taken was mainly pus and then swabs from maxillary sinuses, less frequently swabing from dermal fistulas and wounds with the lowest number of bone swabs (Table 2).

The Swabs were placed in number 1 transport kits and then sent to the Bacteriological Unit of the Central Laboratory at the SPSK-M. The bacteria were identified in a Vitek 2 compact analyser, with GP (for Gram-positive) and GN (for Gram-negative) used. Before a relevant identification card could be used, Gram-stained bacteriological preparations were made. Yeast-like fungi of the* Candida* genus were identified by means of Candida ID bioMérieux chromogenic plates and the Auxacolor 2 test by Bio-Rad.

For clinically significant isolates, antibiograms were made (the disc diffusion or automatic method) using a VITEK 2 compact bioMérieux analyser. Until 31 December 2011, antibiograms for *α*-haemolytic* Streptococcus viridans* and *β*-haemolytic* Streptococcus pyogenes* were made manually on the Müller-Hinton agar with sheep blood using discs by Becton-Dickinson. The media were incubated in thermostats at 35°C for 16–18 hours in a CO_2_ atmosphere.

In the automatic method, antibiograms were made with a Vitek 2 compact analyser using AST-P 534 and AST-533 cards for other streptococci, AST-P 536 for staphylococci, and AST-N 019 AST-N022 for Gram-negative bacteria. Since 1 January 2012, AST-586, AST-576, and ST01 cards have been in use for streptococci, AST-P580 for staphylococci, and AST-N84, AST-N259, AST-N93, and AST-N260 for Gram-negative bacteria.

Antibiogram interpretation is as follows: susceptible, semisusceptible, and of resistance, concerning the disc method. Antibiogram interpretation is as follows: susceptible, semisusceptible, and of resistance and it is defined as MIC (minimum inhibitory concentration or that it is the lowest antibiotic concentration which can inhibit the growth of a given microorganism). It is concerned with antibiograms performed on cards.

### 2.1. Statistical Analysis

The susceptibility of Gram-positive and Gram-negative bacteria has been compared to nine antimicrobial drugs over two periods, 2008–2010 and 2011-2012. The results were subject to statistical analysis using Fischer's test at the significance of *P* < 0.05. A one-way ANOVA was performed with Dunnett's posttest using GraphPad Prism version 5.00 for Windows, GraphPad Software, San Diego, California, USA.

## 3. Results

Among the bacteria isolated from the patients, those Gram-positive bacteria dominated at 541, that is, 74.5%. Gram-negative bacteria were present in 177 (24.4%) cases. Fungi of the* Candida* genus were isolated in eight cases (1.1%). As for the Gram-positive bacteria, streptococci dominated, accounting for 284 strains, most frequently being* Streptococcus mitis *and* Streptococcus oralis*, the number of which has grown considerably over the last two years from seven (9.1%) cultured strains in 2008 to 24 (15.6%) in 2011, and 29 (14.6%) in 2012. Also the growth of* Streptococcus viridans* in 2012 to 26 cultured strains, accounting for 13.1%, is noticeable. In 2008, just three (3.9%)* Streptococcus viridians *were cultured. At the same time, the number of methicillin-sensitive* Staphylococcus aureus* (MSSA) went down from 14 (18.2%) strains in 2008 to eight (4%) in 2012. As for Gram-negative bacteria,* Escherichia coli* (35),* Klebsiella pneumoniae* (33), and* Haemophilus influenzae* (32) dominated (Table 3). Over the last year, there have been more cultured* Enterobacteriaceae *(six strains, i.e., 3%). Over the last two years, the number of cultured fungi has grown too of the* Candida *genus. In 2011-2012, six strains were cultured as compared with the previous three-year period (two strains of those fungi).

In a period of five years, 24 alert pathogens were detected, that is, 3.3% of all the cultured bacterial strains. The highest number (12, i.e., 50% of all the alert pathogens) was detected in 2012 (Table 4). This testifies to a sudden proliferation of antibiotic-resistant strains. In 2008–2011, no strain of a methicyllin-resistant* Staphylococcus aureus* (MRSA) was cultured, while in 2012 there were two (1%) MRSA strains. The 2012 picture looks similar to the alert pathogens* Klebsiella pneumoniae* (Table 3) and* Escherichia coli*, absent before.

**Figure 1:**
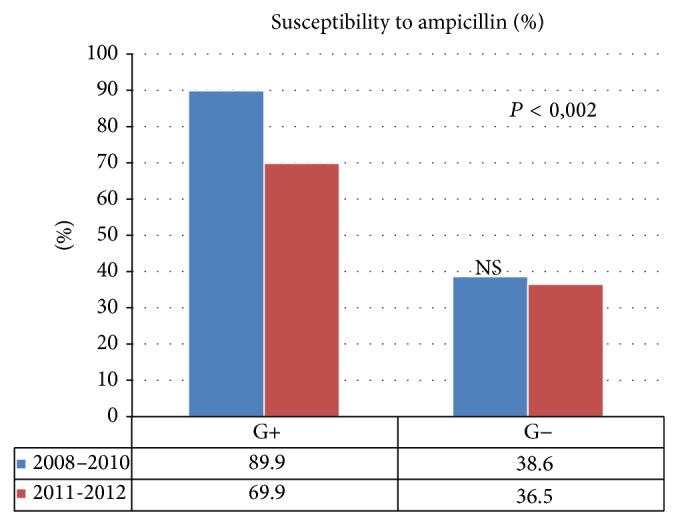
Susceptibility of Gram-positive and Gram-negative bacteria to ampicillin, compared in 2008–2010 and 2011-2012.

**Figure 2:**
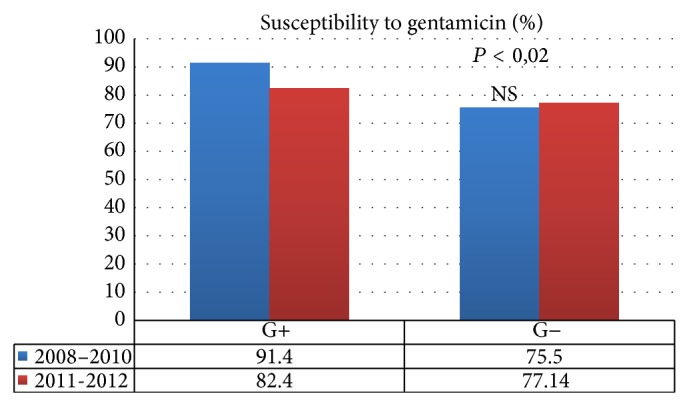
Susceptibility of Gram-positive and Gram-negative bacteria to gentamicin, compared in 2008–2010 and 2011-2012.

Statistically, the difference in the weakened susceptibility of Gram-positive bacteria to ampicillin (*P* = 0.0017) (Figure 1) and gentamicin (*P* = 0.0124) (Figure 2) in a comparison between 2008–2010 and 2011-2012 was significant. There was no statistical significance, however, as regards the susceptibility of Gram-negative bacteria to those antibiotics. No statistical significance was found in terms of the susceptibility of Gram-positive and Gram-negative bacteria to amoxicillin/clavulanate, ciprofloxacin, sulfamethoxazole/trimethoprim, penicillin, vancomycin, imipenem, and clindamycin when the periods of 2008–2010 and 2011-2012 were compared (Table 5).

Noticeably, all the Gram-positive bacteria have become more resistant to all antibiotic groups. The drop was the steepest for ampicillin and imipenem and the flattest for vancomycin: from 100% to 98.8% when the periods of 2008–2010 and 2011-2012 were compared. Clindamycin proved to be of relatively little efficacy, which dropped from 66.8% in 2008–2010 to 61.4% in 2011-2012.

Gram-negative bacteria showed to be more susceptible to ampicillin, ciprofloxacin, and sulfamethoxazole/trimethoprim when the periods of 2008–2010 and 2011-2012 were compared. In turn, less susceptibility was found for amoxicillin with clavulanate, gentamicin, and imipenem.

## 4. Discussion

As more and more bacteria that are resistant and multidrug resistant to antibacterial medication appear while the possibility of making new effective drugs is limited, the common practice of antibiotic use is being discussed and sometimes questioned. The World Health Organisation warns that the fight against hospital-acquired infections including multidrug-resistant bacterial strains is being progressively lost and the planet may find itself on the eve of a postantibiotic era [10].

Baumgartner and Xia, from USA, have assessed antibiotic resistance. The percentages of susceptibility for the 98 species were penicillin V: (85%), (91%); amoxicillin+ clavulanic acid: (100%); and clindamycin: (96%) [11]. We obtained different results: penicillin V: 50%; amoxicillin+ clavulanic acid 62%; and clindamycin 63%.

Rega et al. from USA have demonstrated that the most common bacteria isolated from head and neck space infections of odontogenic origin were* Streptococcus viridans*. The bacteria were found to be 64% G+. Gram-positive cocci were isolated 57.7% of specimens and Gram-negative rods were isolated in 33% of cultures [12]. This contradicts our results where the most commonly isolated microorganism was* Streptococcus mitis and Streptococcus oralis* and we have observed a constant decline of* Streptococcus viridans.* Gram+ bacteria were isolated in 74.5% while Gram− bacteria were isolated in 24.4%.

The most common bacteria isolated by Walia et al. from India were* Staphylococcus aureus*,* Klebsiella*,* Escherichia coli*, and* Peptostreptococcus* [13]. We observed a declining number of these bacteria.

Kedzia et al. isolated bacteria originating from 39 intraoral abscesses. In all the samples, they isolated bacteria and highly rare fungi. Those were mainly anaerobes, Gram-negative bacteria predominantly of the* Prevotella, Bacteroides, *and* Fusobacterium *genera but also* Peptostreptococcus*. Among the aerobes, Gram-positive cocci, mostly* Streptococcus, *were dominant [14]. That does not support our findings, where anaerobes were clearly a minority. From the purulent cultures, mainly Gram-positive pyococci were isolated and mainly also the* Streptococcus *genus. This is probably linked to the incorrect method of sampling for bacteriological testing and keeping the samples for too long before the tests.

The literature of the subject features an increasing number of articles reporting research focusing on whether prophylaxis with antibacterial drugs is absolutely necessary when* Enterococcus* strains (VRE) are becoming dramatically more resistant to vancomycin.* Enterococcus* strains used to be considered pathogens of little clinical relevance, while today they have become responsible for urinary tract infections, endocarditis, bacteraemias, and sepsis. In particular, they cause ill conditions in patients subject to immunosupression. Even the newest antibiotics fail, like linezolid: introduced in 2000 and much hoped for as a cure against the continuously proliferating VRE strains, it proved ineffective already in 2002 against VRE-induced infections in Western Europe. And then there are other alert pathogens like MRSA, the multidrug-resistant* Pseudomonas aeruginosa, Escherichia coli *ESBL, and* Klebsiella pneumoniae* ESBL. In search of new effective antibacterial drugs, Warnke et al. [10] of Australia have proved the efficacy of plant oils from lemongrass (*Cymbopogon*), tea tree, and* Eucalyptus*. Lemongrass oil is particularly active against Gram-positive bacteria while tea-tree oil is active against those Gram-negative bacteria. Such substances cause the degradation of the bacterial cell wall and decrease in osmotic tolerance. Tan et al. of Singapore conducted multicentre randomised clinical trials concerning the use of antibiotics by 329 healthy patients subject to routine implantation treatments. They were assessed for the incidence of pain, oedema, bleeding and lividity for a fortnight after the treatment. The results of comparative studies in four patient groups show that antibiotic prophylaxis both before and after the treatment has no impact on the result of the treatment and postoperative complications. As is known, antibiotics are recommended after implantations [15]. Another article by Adelson and Adapp of New York focuses on taking antibiotics orally by patients with chronic inflammation of the paranasal sinuses. The trial involved using macrolides compared with placebo and did not show any significant improvements in treatment efficacy. The authors place much emphasis on causal treatment searching for odontogenic grounds and their elimination rather than an additional antibiotic therapy. Such an approach is highly commendable and confirmed as right by our practice over the years of treating patients in our clinic. The authors point out the positive impact of a long-term antibiotic therapy with macrolides only in chronic sinusitis patients with lowered levels of immunoglobulin [16]. Lodi et al. of Milan conducted 18 randomised double-blind placebo-controlled trials using antibiotic prophylaxis in 2,456 healthy patients subject to the extirpation of retained third molar teeth. The results showed that, when compared with a placebo, antibiotics possibly reduced the infection risk and the incidence of a “dry socket” by around 70%. However, the study failed to prove that they had an impact on fever, oedema, or trismus up to seven days after the treatment. The authors concluded that in order to prevent a single infection after the extirpation of a retained third molar, twelve patients should take antibiotics [17]. Sisalli et al. (amoxicillin and clavulanic) compared the efficacy and side effects of amoxicillin with clavulanic acid (first-line drug) and those of ceftazidime (second-line drug) in prophylaxis of the extirpation of retained third molars. In 107 patients, in two groups, such antibiotics were administered over five days postoperatively and no statistical significance was found between them. That led to the conclusion that there were no indications for the routine intramuscular administration of second-line antibiotics in prophylaxis after the extirpation of retained third molars. Does this mean more benefits than harm, given the ever-growing resistance of bacteria to antibiotics? At our Clinical Outpatient Unit, antibiotics are indicated only after long surgeries involving the removal of much bone tissue in order to extirpate totally deep-retained third molars. This is due to an enhanced risk of bone inflammation and the “dry socket,” a form of a limited osteitis.

Schaefer and Caterson of Boston conducted a retrospective study of 79 patients treated by osteosynthesis because of mandibular fractures. They compared the effectiveness of antibiotic prevention with ampicillin combined with sulbactam versus clindamycin. It was shown that only 19.35% of the patients treated with clindamycin sustained inflammatory complications against just 7.59% of those treated with ampicillin and sulbactam. The conclusion is that for prophylactic reasons, such antibiotics should be used that act against both aerobic and anaerobic bacteria. Observations from our clinic have made us refrain from administering antibiotics in the case of healthy patients with fresh uncomplicated fractures of the facial skeleton. Antibiotics are indicated for advanced-age patients with systemic illnesses and in old fractures complicated by a purulent inflammation [3].

Meropenem is indicated in empiric treatment prior to the identification of causal microorganisms in therapies of serious infections in both adults and children. Constantinides F. et al. (rapidly progressing) described a case of a rapidly progressing subperiosteal orbital abscess as a complication of pharyngitis caused by Group A *β*-haemolytic* Streptococcus pyogenes* in a healthy 15-year-old patient. The bacterium is thought to be responsible for circa 15–30% cases of acute pharyngitis in children of 5–12 years of age. In the literature, many complications are described where the bacterium is the etiological factor. In our material, it was isolated only sporadically (three cases).

The study shows that clindamycin proves to be of relatively little efficacy against Gram-positive bacteria as its effectiveness dropped to around 61%. That may be related to the fact that the substance is very widely used in dentistry in the form of clindamycin.

As bacteria occurrence is place and time dependent, drug selection must account for the current geographical and epidemiological data [18]. Because of that, this study does not allow us to draw a general conclusion concerning the use of antibiotic agents.

## 5. Conclusions

1. The most often isolated bacteria were* Streptococcus mitis *and* Streptococcus oralis, *whose number has grown over the last two years. The trend can be observed for more streptococci with the exception of the* Viridans *group. At the same time, the numbers for* Staphylococcus aureus *have dropped.
2. Judging by the resistance test results, empiric therapies should be based on ciprofloxacin and gentamicin.
3. It has been observed that all the Gram-positive bacteria are becoming more resistant to all antibiotic groups. The steepest resistance reduction concerned ampicillin and imipenem while the resistance drop was the lowest in the case of vancomycin.

## Figures and Tables

**Table 1:** The number of the patients subject to the examination with gender breakdown.

Gender	Years					Total
	2008	2009	2010	2011	2012	
M	31	63	61	59	70	284
F	23	48	33	47	49	200

Total						484

**Table 2:** Types of swabs taken over the years.

Swab origin	Year				
	2008	2009	2010	2011	2012
Abscess	21	98	73	65	78
Sinus	25	44	20	24	27
Dermal fistula	5	13	17	38	44
Bone	1	—	7	8	11
Wound	9	—	7	12	25
Oral cavity	2	5	4	3	6
Pharynx	11	—	2	4	2
Nose	1	—	—	—	—
Urine	2	—	—	—	—
Blood	—	2	4	—	6

Total	77	162	134	154	199

**Table 3:** Microorganisms isolated from 484 patients treated at the Cranio-Maxillo-Facial Surgery Chair and Clinic and the Clinical Outpatient Unit for Maxillofacial Surgery.

Microorganism	2008	2008%	2009	2009%	2010	2010%	2011	2011%	2012	2012%	Total
Coagulase (−) *Staphylococcus *	7	9.09	33	20.37	47	35.07	41	26.62	38	19.10	166
MRSA *S.aureus *	0	0.00	0	0.00	0	0.00	0	0.00	2	1.01	2
MSSA *S.aureus *	14	18.18	10	6.17	13	9.70	9	5.84	8	4.02	54
Other G (+) cocci	4	5.19	13	1.23	5	3.73	5	3.25	2	1.01	29
*SS. mitis* and *oralis *	7	9.09	11	6.79	11	8.21	24	15.58	29	14.57	82
Other *α*-haemolytic streptococci	10	12.99	21	12.96	16	11.94	16	10.39	26	13.07	89
*Β*-haemolytic streptococci	10	12.99	13	8.02	10	7.46	2	1.30	9	4.52	44
Viridans streptococci	3	3.90	8	4.94		0.00	9	5.84	26	13.07	46
*Enterococcus *	3	3.90	4	2.47	2	1.49	8	5.19	6	3.02	23
Total cocci	58	75.32	113	69.75	104	77.61	114	74.03	146	73.37	535
Other G (+)	0	0.00	2	1.23	1	0.75	1	0.65	2	1.01	6
Total G (+)	**58**	**75.32**	**115**	**70.99**	**105**	**78.36**	**115**	**74.68**	**148**	**74.37**	**541 (74.52%)**
*E. coli *	5	6.49	10	6.17	5	3.73	5	3.25	10	5.03	35
*Klebsiella *	3	3.90	11	6.79	4	2.99	5	3.25	10	5.03	33
*Pseudomonas aeruginosa *	3	3.90	1	0.62	1	0.75	0	0.00	4	2.01	9
Enterobacteriaceae	3	3.90	3	1.85	1	0.75	0	0.00	6	3.02	13
*Haemophilus *	1	1.30	7	4.32	10	7.46	8	5.19	6	3.02	32
*Serratia *	0	0.00	1	0.62	1	0.75	1	0.65	0	0.00	3
*Acinetobacter *	1	1.30	3	1.85	2	1.49	1	0.65	4	2.01	11
*Proteus *	1	1.30	2	1.23	1	0.75	3	1.95	1	0.50	8
*Enterobacter *	0	0.00	5	3.09	4	2.99	5	3.25	4	2.01	18
Other G (−)	1	1.30	3	1.85	0	0.00	8	5.19	3	1.51	18
Total G (−)	**18**	**23.68**	**46**	**35.19**	**29**	**21.64**	**36**	**23.38**	**48**	**22.11**	**177 (24.38%)**
*Candida *	1	1.30	1	0.62	0	0	3	1.95	3	1.51	**8 (1.10%)**

Total microorganisms	**77**	**100.00**	**162**	**100**	**134**	**100**	**154**	**100**	**199**	**100**	**726 (100%)**

G (+): Gram+ bacteria; G (−): Gram− bacteria.

**Table 4:** List of alert pathogens.

Microorganism	Year										Total
	2008	2008%	2009	2009%	2010	2010%	2011	2011%	2012	2012%	
MRSA	0	0	0	0	0	0	0	0	2	1.01	2
*Streptococcus pyogenes *	0	0	2	0	0	0	0	0	1	0.50	3
*Streptococcus pneumoniae *	0	0	0	0	1	0.75	0	0	0	0	1
*Escherichia coli *	0	0	0	0	0	0	0	0	2	1.01	2
*Klebsiella pneumoniae *	0	0	0	0	0	0	0	0	3	1.51	3
*Acinetobacter * spp.	1	1.32	2	1.23	2	1.49	0	0	2	1.01	7
*Pseudomonas* spp.	0	0	1	0.62	1	0.75	0	0	2	1.01	4
*Candida *	1	1.32	0	0	0	0	1	0.65	0	0	2

Total	2		5		4		1		12		24

**Table 5:** Susceptibility of Gram-positive and Gram-negative bacteria to antibiotics in 2008–2010 and 2011-2012.

Antibiotic	Gram-positive			Gram-negative		
	2008–2010	2011-2012	5 years	2008–2010	2011-2012	5 years
Ampicillin	124 (89.9%)	93 (69.9%)	223 (82%)	27 (36.5%)	17 (38.6%)	44 (37.3%)
Amoxicillin clavulanate	N/A	N/A	N/A	42 (64.6%)	28 (60.9%)	70 (63%)
Ciprofloxacin	75 (87.2%)	20 (80%)	95 (85.6%)	60 (87%)	25 (89.3%)	85 (87.6%)
Sulfamethoxazole/ trimethoprim	139 (91.4%)	139 (82.4%)	275 (86.7%)	40 (75.5%)	54 (77.14%)	94 (74%)
Gentamicin	105 (94.6%)	86 (84.3%)	191 (89.7%)	51 (94.4%)	56 (87.5%)	107 (90.7%)
Vancomycin	248 (100%)	260 (98.8%)	508 (99.4%)	N/A	N/A	N/A
Imipenem	22 (100%)	30 (83.3%)	52 (89.6%)	69 (92%)	5 (90%)	123 (91.1%)
Clindamycin	171 (66.8%)	148 (61.4%)	319 (64.2%)	N/A	N/A	N/A
Penicillin	135 (53.4%)	119 (50.4%)	254 (51.9%)	N/A	N/A	N/A

N/A: not applicable.
